# Antiplatelet Therapy in Coronary Artery Disease: A Daunting Dilemma

**DOI:** 10.3390/jcm7040074

**Published:** 2018-04-09

**Authors:** Surya Chaturvedula, Daniel Diver, Aseem Vashist

**Affiliations:** 1 Department of Cardiology, University of Connecticut School of Medicine, Farmington, CT 06030, USA; chaturvedula@uchc.edu (S.C.); ddiver@stfranciscare.org (D.D.); 2 Hoffman Heart and Vascular Institute at St Francis Hospital and Medical Center, Hartford, CT 06105, USA; 3 VACT Healthcare System, West Haven, CT 06516, USA

**Keywords:** dual antiplatelet therapy, coronary artery disease, acute coronary syndrome, drug eluting stent, bare metal stent, aspirin, clopidogrel, prasugrel, ticagrelor, triple therapy, oral anticoagulants

## Abstract

Percutaneous coronary intervention (PCI) with stenting for the treatment of acute coronary syndrome (ACS) is the contemporary standard of care. Such treatment is followed by dual antiplatelet therapy (DAPT) comprising of aspirin and a P2Y12 inhibitor. The efficacy of this therapy has been well established but the optimal duration of DAPT remains elusive, and has thus far attracted a prodigious deal of scientific attention. The decision regarding DAPT duration can be clinically challenging in the modern era with the evolution of newer stents, more potent antiplatelet agents, and novel anticoagulant drugs in addition to an older patient population with multiple comorbidities. Major societal guidelines have emphasized comprehensive assessment of ischemic and bleeding risk, in turn recommending individualization of DAPT duration, thus encouraging “shared decision making”. The following review is aimed at critically evaluating the available evidence to help make these crucial clinical decisions regarding duration of DAPT and triple therapy.

## 1. Introduction

Coronary revascularization with percutaneous intervention (PCI) is currently the standard of care in the treatment of patients with acute coronary syndrome (ACS), and has become one of the most frequently performed therapeutic procedures in Medicine [1]. Dual antiplatelet therapy (DAPT) comprising of aspirin and a P2Y12 inhibitor is one of the most commonly prescribed therapies in cardiovascular medicine. The optimal duration of DAPT after stent implantation has been a matter of intense debate and has attracted a great deal of scientific attention. As we celebrate the various advances in the techniques and technology of transcatheter therapeutics in this 40th year of Interventional Cardiology, the optimal duration of DAPT continue to be elusive. Most recent transatlantic guidelines have called for the comprehensive assessment of ischemic and bleeding risks thus emphasizing individualization of DAPT [2,3]. The following review is aimed at critically evaluating the available evidence to help make crucial clinical decisions regarding duration of DAPT and triple therapy.

## 2. Evolution of PCI and DAPT

Careful examination of the history of PCI provides important insights into the evolution of DAPT [4]. Initial enthusiasm of angioplasty was significantly limited by vessel closure due to recoil, dissections and restenosis thus leading to development of stents to offer luminal integrity without compromising safety [5,6]. Bare metal stents (BMS) were affected by restenosis with a need for repeat revascularization in up to one-third of the patients [7] besides early stent thrombosis (EST; <30 days) [8]. Drug eluting stents (DES) were clearly superior to BMS in reducing restenosis and rates of repeat revascularizations [9,10,11,12]. There was a trend for increased late stent thrombosis (LST) (>30 days, <1 year) and very late stent thrombosis (VLST) (>1 year) in the first generation DES. Subsequently, second generation DES endowed with better biocompatibility and thinner platforms ensured improved vessel healing [13]. In a meta-analysis of four randomized controlled trials (RCTs) comparing Everolimus eluting stents (second generation DES) vs. Paclitaxel eluting stents (first generation DES) a significant reduction in stent thrombosis (ST) was noted (0.7% vs. 2.3%; odds ratio (OR): 0.32; *p* < 0.00001) [14].

The idea of antiplatelet therapy in reducing thrombosis following PCI was kindled three decades ago [15]. However, use of DAPT became a standard in BMS era to reduce the rates of EST [16]. DAPT was reserved for 3–6 months for the use of first generation DES to prevent ST and to ensure endothelialization [9,10,17]. With the realization of higher thrombotic milieu in the first year with these stents, especially with interruption of DAPT [18,19], consensus based guidelines recommended prolonging DAPT to up to 12 months [20]. This philosophy was reinforced by the observed benefits of such therapy in prevention of atherothrombosis of nonstented segments in coronary vasculature [21]. With the ubiquitous use of second generation DES with lower rates of ST, and evidence from multiple RCTs supporting shorter DAPT, guidelines proposed optimal DAPT duration of 6–12 months [2,22]. Other important considerations guiding decisions regarding duration of DAPT are discussed in this review and include the complexity of the procedure (e.g., bifurcation stenting, Chronic total occlusion), location of PCI (e.g., left main PCI) and the type of stent used (newer generation of stents with thinner struts and biocompatible polymers provide a favorable profile compared to the first-generation stents). The trade-off regarding ischemic and bleeding events was studied in two large RCTs, which demonstrated benefit in reducing stent-related and unrelated ischemic events at the cost of increased bleeding [23,24] thus reigniting the short vs. long debate. The most recent iteration of guidelines takes cognizance of all these data and call for shared decision making and individualizing DAPT [3].

## 3. Antiplatelet Agents and Their Landmark Trials

The cardinal pharmacological properties of various P2Y12 inhibitors are enumerated in Table 1. We also highlight important differences in the recommended periods of discontinuation of these agents in the lead up to non-emergent surgery.

While an exhaustive review of all the trials is beyond the scope of this review, Table 2 summarizes the evidence leading to the inception of various antiplatelet agents in the treatment of coronary artery disease (CAD). The outcome measures observed in these trials highlight the ability of these drugs in improving cardiovascular outcomes, albeit at the cost of increasing bleeding.

The benefits of DAPT in a RCT setting were first seen in the *CURE* [27] trial in which combination of aspirin and clopidogrel in comparison with aspirin monotherapy was assessed in patients undergoing PCI in myocardial infarction (MI) without ST segment elevation. 12,562 patients were enrolled and at mean follow-up of 9 months, DAPT was associated with a significant reduction in the composite primary endpoint of cardiovascular mortality (CVM), nonfatal MI, or stroke (9.3% vs. 11.4%, Relative risk (RR): 0.80; *p* < 0.001; Number needed to treat (NNT) = 48). However, this came at a cost of increased rate of major bleeding (3.7% vs. 2.7%, RR: 1.38; *p* = 0.001). This trial was instrumental in establishing the 12 months of DAPT as standard of care in the treatment of ACS patients.

In *TRITON-TIMI 38* [30] trial, 13,608 patients with ACS awaiting PCI were randomized to either prasugrel or clopidogrel in addition to the usual care. At mean follow-up of 14.5 months, composite primary endpoint of CVM, nonfatal MI, or stroke was significantly lower in the prasugrel group (9.9% vs. 12.1%, HR: 0.81; *p* < 0.001; NNT = 46). These benefits came at the cost of increased risk of bleeding. Major bleeding was higher with the use of prasugrel group vs. clopidogrel (2.4% vs. 1.8%, HR 1.32; *p* = 0.03). Also, greater in the prasugrel group was the rate of life-threatening bleeding (1.4% vs. 0.9%; *p* = 0.01), including nonfatal bleeding (1.1% vs. 0.9%; HR 1.25; *p* = 0.23) and fatal bleeding (0.4% vs. 0.1%; *p* = 0.002). There was no significant difference in either CVM or all-cause mortality (ACM). Interestingly, the benefits appeared within days from randomization and persisted beyond the first year. In the sub-group analysis of patients with ST elevation MI (STEMI), there was an even greater benefit in the primary outcome (6.5% vs. 9.5%; HR: 0.68; *p* = 0.0017) without the incremental bleeding risk [35]. In the subsequent TRILOGY ACS trial [32], there was no significant risk reduction of primary endpoint with the use of prasugrel in patients with unstable angina (UA) and non-ST elevation myocardial infarction (NSTEMI) treated without revascularization.

In one of the largest RCTs’, Ticagrelor was compared to clopidogrel in the *PLATO* trial [31]. PLATO randomized 18,624 patients with ACS (37.5% presenting with STEMI) were randomized to ticagrelor or clopidogrel in addition to standard care. At 12 months, ticagrelor group had lower composite primary outcome of CVM, MI, or stroke (9.8% vs. 11.7%; HR: 0.77–0.92; *p* < 0.001) and there was insignificant increase in major bleeding (11.6% vs. 11.2%; HR: 1.04; *p* = 0.43). A reduction in vascular mortality (4% vs. 5.1%; HR: 0.79; *p* < 0.001) and ACM (4.5% vs. 5.9%; HR: 0.78; *p* < 0.001) were also noted. However, the reduction in stroke was statistically not significant (1.5% vs. 1.3%; HR: 1.17; *p* = 0.22). Ticagrelor is the only antiplatelet agent shown to decrease the ACM compared to clopidogrel, though given the hierarchical statistical design of this study, the significance of this finding is attenuated.

The real-life experience of ticagrelor was evaluated in *SWEDEHEART* registry [36]. This nonrandomized prospective cohort study of 45,073 ACS patients in Sweden demonstrated amplified benefits of ticagrelor in comparison with clopidogrel. The composite primary outcome of ACM, readmission with myocardial infarction (MI) or stroke, was lower with ticagrelor group (11.7% vs. 22.3%, adjusted HR 0.85). In a subset of patients undergoing PCI on ticagrelor, the PCI-related in-hospital bleeding was higher (3.7% vs. 2.7%, adjusted OR: 1.57 (1.30–1.90)). This registry data certainly corroborates the evidence from PLATO trial but some major differences are noteworthy, as evidenced by the mean age of patients in the present study being 8 years higher (70 vs. 62 years) and a higher proportion of patients with history of stroke (10.8% vs. 3.9%) and heart failure (10.3% vs. 5.6%).

## 4. Duration of DAPT

Traditionally 12 months of DAPT duration has been considered as the standard, with 3 months and 6 months of DAPT representing short DAPT (S-DAPT) and >12 months representing longer DAPT (L-DAPT) durations. The conception of S-DAPT was to reduce bleeding without compromising the safety and efficacy of PCI, while L-DAPT was tested with a hope to improve stent-related and stent-unrelated ischemic (atherothormbotic) events. With the development of better stent platforms amounting to reduction in rates of ST and restenosis paralleled by development of potent antiplatelet agents, the “optimal” duration of DAPT has been extensively evaluated but still remains elusive. This constant dualistic debate of “short” vs. “long” has certainly lead to significant uncertainty and confusion among the treating providers. Some observers have recommended an end to such a dogmatic approach laced with academic debates, and emphasized shared decision-making and individualization of therapy [37].

## 5. Evidence on DAPT Duration

### 5.1. RCTs’

To date, there have been several RCTs’ and several meta-analyses of these trials to evaluate for optimal duration of DAPT. At the outset, it is crucial to note the several limitations to these trials including, but not limited to, flaws in design ultimately leading to lack of power in detecting difference in hard endpoints, varying patient and lesion complexity, diverse clinical settings, low event rates, different times of randomization, slow enrollment, dissimilar endpoints, and differential use of stents (BMS; first vs. second-generation DES), thus making comparable interpretation difficult and yielding inconsistent results [38].

#### 5.1.1. RCT with S-DAPT

There have been 12 RCTs’ using S-DAPT to determine its relative efficacy in preventing major adverse cardiovascular and cerebrovascular events (MACCE) including ST, and to determine the relative safety of such DAPT duration for major bleeding in comparison with standard or L-DAPT. Unfortunately, none of these trials were independently powered to evaluate the rates of safety endpoint of ST, which is infrequent.

#### 5.1.2. S-DAPT vs. Standard DAPT Duration

The hypothesis of noninferiority of S-DAPT to standard care was tested in 9 RCTs. These are comparatively summarized in Table 3.

It is imperative to note that patients were randomized to DAPT duration at the time of stent implantation in all trials except in ISAR-SAFE [43]. There is significant heterogeneity among these trials with regards to the enrollment of patients with ACS, diabetes, and the type of stent used.

The *ISAR-SAFE* [43] trial was actually designed to enroll 6000 patients with a noninferiority hypothesis. However, it was prematurely terminated after enrolling 4000 patients’ due to slow enrollment but still achieved noninferiority. These patients who had undergone PCI with DES were randomized at 6 months to interrupt or continue 12 months of DAPT. There was a fair representation of patients with ACS (40%), with 10% suffering from STEMI and 30% with multivessel CAD. Second-generation DES were predominantly used (89%) and mostly used in treatment of single lesion (63%). Primary composite outcome of death, MI, ST, stroke, or thrombolysis in myocardial infarction (TIMI) major bleeding occurred in 1.5% of patients on S-DAPT and 1.6% with standard DAPT (*p*_ni_ < 0.001 with predefined noninferiority margin of 2%). Both groups had similar rates of TIMI major bleeding.

Two trials with a novel approach were presented at Transcatheter Cardiovascular Therapeutics meeting (TCT 2017), Denver, CO, 1 November 2017. In the *DAPT-STEMI* trial [46], patients with STEMI and undergoing primary PCI with a second-generation DES (zotarolimus-eluting stent (ZES)) were randomized in a 1:1 fashion to receive either 6 months (*n* = 433) or 12 months of DAPT (*n* = 437) to assess the safety and efficacy of such DAPT durations. Importantly, patients without any events in the first 6 months (MI, ST, target vessel failure (TVF) or target lesion failure (TLF), or stroke/bleeding requiring DAPT discontinuation) were included in the analysis. Patients requiring left main coronary artery (LMCA) intervention were excluded. All three contemporary P2Y12 agents were used (clopidogrel: 42%, prasugrel: 30%, ticagrelor: 29%).The primary outcome, ACM, MI, revascularization, stroke, and TIMI major bleeding at 18 months was lower in 6-month vs. 12-month DAPT (4.8% vs. 6.6%, *p*_ni_ = 0.004). Although, this 2-year outcome data establishes noninferiority of S-DAPT in STEMI patients, this trial was not powered to evaluate for individual safety endpoints. Long-term data would be crucial before such short DAPT duration is adapted into clinical practice in the treatment of ACS.

In the REDUCE trial [47], 3-month vs. 12-month DAPT was assessed for safety and efficacy after implantation of a bioabsorbable polymer-based metallic sirolimus eluting stent with a luminal CD34+ antibody coating in patients with ACS. The rationale behind testing such a stent was to use the combination of abluminal release of sirolimus (to prevent neointima formation), and capture of endothelial progenitor cells (to enhance stent re-endothelialization). The cumulative survival, free from the primary study endpoint of ACM, MI, ST, stroke, TVR, or bleeding for 3-month vs. 12-month DAPT, was 91.7% vs. 91.5%, *p*_ni_ < 0.001. There were, however, concerning safety signals with a higher risk of ACM (1.9% vs. 0.8%, *p* = 0.07) and ST (1.2% vs. 0.4% *p* = 0.08), with shorter duration of DAPT with no difference in bleeding. Cautious interpretation of these results suggests that though the noninferiority hypothesis was met, the margin of noninferiority was quite generous, and the trend of some ischemic endpoints impoverished the 3-month DAPT group. 

#### 5.1.3. S-DAPT vs. L-DAPT

Three RCT till dates have been published as summarized in Table 4.

The implications of the data from these trials is certainly influenced by the heterogeneity of patients and of the stents used. These trials were powered to look for difference in bleeding, and due to the low ischemic event rates, any conclusions drawn to qualify the efficacy would be inaccurate. More recently, *NIPPON* trial [50] was performed in Japan using bioabsorbable polymer-based DES. This trial tested for noninferiority of 6-month DAPT vs. 18 month DAPT, and randomized 3775 patients. The composite primary outcome of ACM, MI, stroke, and major bleeding was similar (1.92% vs. 1.45%) thus meeting the noninferiority. However, the margin for such noninferiority was set wide at 2% which exceeded the event rate of the experimental arm and the study was prematurely terminated thus raising concerns and these results should be judiciously interpreted.

#### 5.1.4. Standard DAPT vs. L-DAPT

The hypothesis of superiority of L-DAPT in reducing the VLST and other ischemic events in comparison with standard DAPT was tested in four RCTs’. These are comparatively represented in Table 5. In all these trials, event-free patients on 1 year of DAPT were randomized to single antiplatelet therapy (SAPT) vs. continuation of DAPT with clopidogrel or prasugrel for varying periods of time.

The *DAPT* [23] trial deserves a special mention for being the only trial which was adequately powered for safety and efficacy endpoints, and also providing some significant insights into L-DAPT. In this trial, 9961 patients who were event free after 12 months’ of DAPT and compliant to DAPT were randomized to continue DAPT for 30 months’ vs. SAPT (with aspirin). About 26% of the participants had ACS and importantly, 47% of the patients received Everolimus eluting stents (EES) and only clopidogrel (65%) and prasugrel (35%) were used as a part of DAPT. In the DAPT group, there was 1% lower VLST and 1.6% fewer MACCE events driven by 2% reduction in rates of MI. These benefits came at a cost of 0.9% absolute increase in moderate to severe GUSTO (Global Utilization of Streptokinase and t-PA for Occluded Coronary Arteries) bleeding and 2.6% increase in BARC (Bleeding Academic Research Consortium) 2, 3, or 5 bleeding. At 33 months’ follow-up, ACM was higher in the DAPT group (2% vs. 1.5%, *p* = 0.052). This increase was attributable to bleeding, trauma, and cancer [54].The authors also interpreted this finding as being due to chance and later it was noted that at baseline, a greater number of patients with a prior history of cancer had been randomly allocated to extended DAPT duration group thus explaining 7 of the 26 deaths in that group. Food and drug administration(FDA) passed a revision refuting an association of increased mortality with extended use of clopidogrel [55]. However, such an increase in fatalities were also observed in other studies [56] with other agents.

More recently, the hypothesis of 48 months of DAPT with clopidogrel being superior to 12 months of DAPT was tested in *OPTIDUAL* [53]. The enrollment was prematurely stopped in this trial. Superiority of L-DAPT could not be established as the composite primary endpoint of death, MI, stroke, or major hemorrhage was similar in both the arms (5.8% vs. 7.5%; HR 0.75; *p* = 0.17). The safety endpoint of moderate and severe GUSTO bleeding (1.9% vs. 1.7%) and BARC 2, 3, or 5 bleeding (2.6% vs. 2.9%) were similar in both groups.

#### 5.1.5. Other RCTs with DAPT Duration

Three other RCTs are worthy of review as they deal with safety and efficacy of DAPT in varied clinical settings.

In the *CHARISMA* [57] (Clopidogrel for High Atherothormbotic Risk and Ischemic Stabilization, Management, and Avoidance) trial, 15,603 patients with cardiovascular risk factors or a history of vascular disease were randomized to receive DAPT with aspirin and clopidogrel vs. SAPT with aspirin. The composite primary endpoint of MACCE at 28 months was similar in both groups (6.8% vs. 7.3%, *p* = 0.22), and there was no significant difference in major bleeding. However, there was 1% risk reduction of MACCE in DAPT group vs. SAPT (6.9% vs. 7.9%; RR 0.88; *p* = 0.046) when analyzed in the pre-specified group of patients with established cardiovascular disease [58]. Patients with prior MI, stroke, or symptomatic peripheral arterial disease (PAD) derived significant benefit from DAPT (7.3% vs. 8.8% HR: 0.83, *p* = 0.01) and there was no significant difference in the rate of severe bleeding (1.7% vs. 1.5%, HR: 1.12; *p* = 0.50); moderate bleeding was significantly increased (2.0% vs.1.3%, HR:1.60; *p* = 0.004) [58].

In *PEGASUS-TIMI 54* [24] (Prevention of Cardiovascular Events in Patients with Prior Heart Attack Using Ticagrelor Compared to Placebo on a Background of Aspirin-Thrombolysis in Myocardial Infarction 54) trial; 21,162 patients with a prior history of MI in preceding 1–3 years, were randomized in a double blinded regimen in 1:1:1 into three groups. This trial was designed to test the efficacy of DAPT (Ticagrelor 90 mg twice daily or 60 mg twice daily) with aspirin vs. SAPT with aspirin. There was 1.2–1.3% absolute risk reduction (ARR) of MACCE events in DAPT groups’ vs. SAPT at the cost of 1.2–1.5% increase in major bleeding. However, there was no excess in fatal bleeding or intracranial hemorrhage. The subgroup analysis of higher risk patients demonstrated more robust benefits. In the diabetic subgroup, there was an ARR of 1.5% (*p* = 0.03) [59]. Patients with prior vascular disease demonstrated a higher event rate, and despite an increased bleeding risk, there was a nearly 5% ARR [24] of ischemic events. Patients with renal disease also had higher event rate but drew more benefit from DAPT therapy with ARR 2.7% [60] of ischemic events.

These two trials underpin the ischemic benefit derived from L-DAPT especially in higher risk patients, albeit at the cost of increased bleeding risk. However, it is noteworthy that a majority of these patients had a period of interruption in DAPT after their initial ischemic event. In fact, in PEGASUS prespecified subgroup analysis, patients with discontinuation period of 1 year or longer before reinitiation of DAPT did not derive any benefit [61].

In the recently published *SENIOR* trial [62], 1200 elderly patients (≥75 years of age) with CAD, were randomly assigned to DES or BMS after an intended duration of DAPT (1 month for stable CAD, 6 months for ACS). There was significant reduction in primary composite endpoint of ACM, MI, stroke, ischemia driven target lesion revascularization in DES vs. BMS (16.4% vs. 11.6%, *p* = 0.016; RR 0.71) thus yielding NNT = 21. This difference was mainly driven by ischemia driven target lesion revascularization (1.7% vs. 5.9%, *p* = 0.0002). Net clinical benefit encompassing MACCE and BARC 2–5 bleeding was significantly lower in DES vs. BMS (14.4% vs. 19.2%, *p* = 0.0239; RR 0.75). Interestingly, ST was low and not different between the groups (0.5% vs. 1.4%, *p* = 0.12). It has to be noted that the aim of this study was to compare the type of stents but not the DAPT duration. However, it provides valuable information in this group that has not been well-represented in prior RCTs’.

### 5.2. Meta-Analyses

The idea of net clinical benefit for the individual patient becomes complicated due the fact that these trials demonstrate reduced ischemic events and increased bleeding with prolongation of DAPT, although with a possible interaction with stent type. This generated a need for meta-analysis of these RCTs’. Many meta-analyses have been performed till date and they have differed significantly in the number of RCTs’ included and also their designs [63,64,65,66,67,68].

In the largest meta-analysis till date [65] including 14 RCTs’ involving 69,644 patients with ACM as the only primary endpoint, there was no significant difference in mortality with L-DAPT in comparison with S-DAPT (HR = 1.05, 95% credible interval, 0.96–1.19). However, since this analysis included mixed populations, moderate heterogeneity was present (I^2^ = 27%) for the treatment effects. In a recent meta-analysis [69] of five RCTs’ with mean follow up of 2 years or longer involving 20,000 patients, S-DAPT was compared to L-DAPT. The primary endpoint was ST and secondary endpoints were ACM, CVM, MI, TVR, TIMI major bleeding and stroke. Compared to L-DAPT, S-DAPT was associated with higher MI (OR 1.48). There were no significant differences between groups in all other endpoints.

## 6. Current Guidelines

The current transatlantic guidelines on DAPT usage are summarized in Table 6.

## 7. Individualization of Therapy

Decisions regarding DAPT duration are complex and they epitomize the current era of “personalized medicine”. The net clinical benefit should be the ultimate goal of this shared decision. DAPT trial offers a decent outlook into this aspect.

In evaluation of net clinical benefit in the DAPT study participants, NNT for benefit from ischemic events was 100 based on 1% ARR for ST; NNT = 50 based on ARR 2% for MI. The number needed to harm (NNH) was 111 based on 0.9% absolute risk of increase (ARI) in bleeding with L-DAPT thus favoring such a strategy [23]. However, when a similar exercise is carried out for the pre-specified patients with second generation DES; NNT = 200 based on ARR 0.5% for ST; NNT = 91 based on ARR 1.1% and NNH = 83 based on ARI 1.2% due to bleeding thus disfavoring L-DAPT for prevention of MACCE rate and mortality [70].

**Figure 1:**
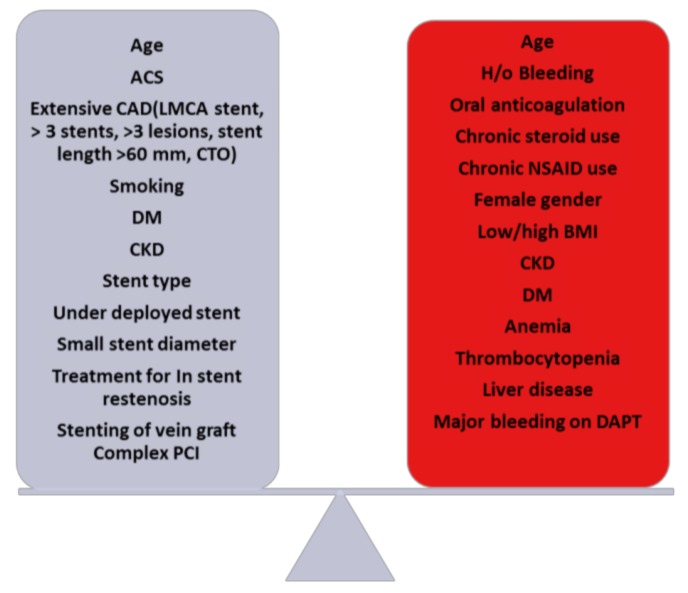
Balance between ischemic and bleeding risks [38,72]. ST = Stent thrombosis; ACS = Acute coronary syndrome; DM = Diabetes mellitus; CKD = Chronic kidney disease; PCI = percutaneous coronary intervention; NSAID = Non-steroidal anti-inflammatory drugs, DAPT = Dual antiplatelet therapy.

Hence, it’s imperative to evaluate the factors conferring ischemic and bleeding risks as listed in Figure 1. Though use of DAPT in reducing ischemic events [24,28] is well known, it is crucial to recognize the increased risk of bleeding with such therapy [24,27] which ultimately has adverse prognostic implications [71] as well.

Risk calculators, as endorsed by the most recent guidelines can be an instrumental in making decisions regarding the duration of DAPT [3]. These are summarized in Table 7.

DAPT score was developed to aid clinicians and patients in the assessment of ischemic and bleeding risks. Since the tool was developed from DAPT study data, it can only be applied to patients completing 12 months DAPT uneventfully. This score was internally validated in DAPT study with moderate discrimination (C statistic, 0.70; 0.68) and calibrated for both ischemia and bleeding risks (goodness-of-fit *p* = 0.81, *p* = 0.34) [72]. This tool was externally validated in the *PROTECT* (Patient Related Outcomes with Endeavor versus Cypher stenting) trial cohort [73].

*PRECISE-DAPT* (Predicting bleeding Complications In patients undergoing Stent implantation and subsequent Dual Anti Platelet Therapy) study group developed a tool from eight RCTs’. The predictive performance of this tool was assessed in the derivation cohort and validated in 8595 patients from the *PLATO* trial and 6172 patients from *BernPCI* registry. In comparison with PARIS bleeding score, this tool demonstrated good discrimination and net reclassification of patients [74].

These tools have not yet been tested prospectively in a RCT setting, and are by no means perfect or substitutive to clinical judgement [75].

## 8. Triple Therapy

Triple therapy refers to the use of oral anticoagulant (OAC) and DAPT. CAD is a common comorbid condition in patients with atrial fibrillation (AF), and its prevalence was reported as 60–65% in Medicare beneficiaries [76]. Guidelines recommend assessment of stroke risk by CHA_2_DS_2_VASc score in patients with AF, and for scores ≥1–2, oral anticoagulant(OAC) is recommended to mitigate risk of thromboembolism commonly manifested as stroke [76,77]. An estimated 5–10% patients undergoing PCI have concomitant AF with a need for OAC [78]. Other clinical situations requiring triple therapy is in patients needing PCI, and with indications for anticoagulation for conditions like deep vein thrombosis(DVT)/pulmonary embolism (PE), mechanical heart valve, left ventricular thrombosis etc. Such therapy comes at a cost of excessive bleeding risk [79]. With the inception of direct oral anticoagulants and potent P2Y12 inhibitors, clinical decisions on triple therapy remain controversial in the ability to optimize the balance between prevention of stroke and ST without unduly increasing bleeding risk.

The following Table 8 summarizes the salient findings from most recent RCTs.

In the *WOEST* trial [80], the warfarin was evaluated and 70% patients had AF as the indication for OAC, while 25–30% had ACS at presentation. The results demonstrated the superiority of dual therapy with warfarin and clopidogrel vs. triple therapy on account of significant reduction in the primary outcome which was any bleeding within 1 year of PCI (19.5% vs. 44.4%; HR 0.36, *p* < 0.001) as well as reduction in ACM (2.5% vs. 6.4%; *p* = 0.027). The heterogeneity of patients with various indications for OAC is a limitation of this study.

Subsequently, with the introduction and prevalent use of direct oral anticoagulants, *PIONEER AF-PCI* [81] used rivaroxaban and *REDUAL-PCI* [82] used dabigatran to evaluate the safety and efficacy of triple therapy exclusively in AF patients undergoing PCI.

In the PIONEER AF-PCI [81], there was 1:1:1 randomization of patients to receive low-dose rivaroxaban (15 mg daily) + P2Y12 inhibitor for 12 months; very low dose rivaroxaban (2.5 mg twice daily) + DAPT for 1, 6, or 12 months or standard therapy with dose adjusted warfarin + DAPT for 1, 6, 12 months per guideline recommended DAPT duration based on the indication and stent type. There was less bleeding in the rivaroxaban groups vs. warfarin (17.4% vs. 26.7%, HR 0.61; *p* < 0.001) without significant difference in MACCE. The rivaroxaban groups had lower re-hospitalization rates in comparison to warfarin ((34.1% vs. 41, 5%, HR: 0.77, *p* = 0.05); (31.2% vs. 41.5%, HR: 0.74, *p* = 0.01)). This trial establishes supremacy of rivaroxaban over warfarin in reducing bleeding and re-hospitalizations but it was criticized for the use of 15 mg dose of rivaroxaban which is not approved for use in AF. It has to be emphasized that since the huge majority of the patients received clopidogrel (95%), this data cannot be extrapolated to the use of other newer and more potent P2Y12 inhibitors as a part of triple therapy regimens.

The results from *REDUAL-PCI* [82] were presented at the American Heart Association’s Annual Scientific Sessions (AHA 2017), Anaheim, CA, 14 November 2017. In this trial, AF patients undergoing PCI were randomized in 1:1:1 fashion to dual therapy with dabigatran at a dose of 110 mg (*n* = 981) vs. dual therapy with dabigatran at a dose of 150 mg (*n* = 763) vs. triple therapy with warfarin (*n* = 981). In the dual therapy group, participants received clopidogrel or ticagrelor in addition to one of two doses of dabigatran. In the triple therapy group, participants received aspirin plus clopidogrel or ticagrelor in addition to warfarin. The duration of aspirin was 1 month after a BMS and 3 months after a DES. About 52% patients had ACS, 82% received DES and 10% received ticagrelor as the P2Y12 inhibitor. The primary safety outcome, incidence of major or clinically relevant non-major bleeding events was lower in both dabigatran groups vs. triple therapy ((15.4% vs. 26.9%, *p*_ni_ < 0.001); (20.2% vs. 25.7%, *p*_ni_ < 0.001)). TIMI major bleeding was also lower in dual vs. triple therapy groups. The primary efficacy outcome, incidence of death, MI, stroke, systemic embolism, or unplanned revascularization occurred in 13.7% of both dual therapy groups vs. 13.4% of the triple therapy group (*p*_ni_ = 0.005). In the sub-group analysis of patients with ACS (52%), ticagrelor was associated with higher bleeding compared to clopidogrel, with and without dabigatran. 

There is also an emerging interest in evaluation of the efficacy of combination therapy with OAC and single antiplatelet agent in improving clinical outcome. In the recently published *COMPASS* [83] trial, in patients with stable CAD, addition of rivaroxaban to aspirin lowered major vascular events (4% vs. 6%; HR: 0.74, 95% CI 0.65–0.86, *p* < 0.0001), but increased major bleeding (3% vs. 2%; HR 1.66, *p* < 0.0001). There was no significant increase in intracranial bleeding or other critical organ bleeding. There was also a significant net benefit in favor of rivaroxaban plus aspirin and deaths were relatively reduced by 23%. Thus, addition of rivaroxaban to aspirin has the potential to substantially reduce morbidity and mortality from CAD. In this trial, after a 30-day run in period, patients were randomly assigned (1:1:1) to receive rivaroxaban (2.5 mg orally twice a day) plus aspirin (100 mg once a day), rivaroxaban alone (5 mg orally twice a day), or aspirin alone (100 mg orally once a day). These doses of rivaroxaban are not available in USA for routine use and the data on such combination therapy is still evolving.

Triple therapy is a clinically challenging situation where in the bleeding risk is enhanced by the combination of DAPT and OAC. The overarching goal is to create a regimen that reduces bleeding risk while maintaining efficacy in reducing the ischemic events. Contemporary regimens include triple therapy with OAC, P2Y12 inhibitor (usually clopidogrel) and aspirin for a variable duration from 1–6 months post PCI depending on the ischemic/bleeding ration followed by cessation of aspirin therapy. The aforementioned several lines of evidence now suggest that it is safe to treat patients who undergo PCI with anticoagulation and clopidogrel monotherapy.

The optimal initial treatment regimen for patients presenting with ACS and high-risk disease is unclear, but the subgroup analysis performed in the *RE-DUAL PCI* trial [82] suggests that patients with ACS who are at high risk for bleeding may be able to tolerate this type of therapy as well. More research is required to help clinicians determine the optimal duration and treatment regimen of DAPT and OACs for this complex group of patients.

The most recent iteration of ESC guidelines provides evidence based recommendations and possible regimens as listed in the Table 9.

## 9. Conclusions and Future Directions

The data on the duration of DAPT in patients with CAD continues to evolve especially with the availability of newer stent designs and potent antiplatelet agents and newer oral anticoagulants. Novel DES has been shown to be safer than BMS in terms of device related adverse events with both standard DAPT [84] and S-DAPT [85]. Prolonged DAPT reduces the ischemic events at the cost of bleeding risk, which continues to accrue with longer duration of DAPT. Thus, optimal duration of DAPT remains a moving target. For our readers, we have summarized the future and emerging trials in Table 10.

The old adage, “there is no free lunch”, aptly applies to this clinical dilemma and therefore decisions on DAPT duration require an astute understanding of both the patient’s ischemic as well as bleeding risks and “shared decision-making” with the patient is recommended.

## Figures and Tables

**Table 1:** Comparative properties involving oral P2Y12 inhibitors.

	Clopidogrel	Prasugrel	Ticagrelor
Binding	Irreversible	Irreversible	Reversible
Onset of action	2–6 h	30 min	30 min
Half-life of active metabolite	30–60 min	30–60 min (distribution) 2–15 h (elimination)	7–9 h
Duration of effect	3–10 days	7–10 days	3–5 days
Frequency of administration	Once daily	Once daily	Twice daily
Discontinuation prior to non-acute surgery	At least 5 days	At least 7 days	At least 3 days

N/A = not applicable. In patients with ACS previously exposed to clopidogrel, switching to ticagrelor is recommended early after hospital admission at a loading dose of 180 mg irrespective of timing and loading dose of clopidogrel, unless contraindicated (IB). All other switching between P2Y12 inhibitors may be considered in cases of side effects/intolerance (IIb-C) [3].

**Table 2:** Landmark trials of antiplatelet agents.

Year	Trial	Drug	Outcome
1988	ISIS-2	Aspirin	Aspirin became the mainstay of therapy in ST elevation myocardial infarction (STEMI)
1996	CAPRIE	Clopidogrel	Clopidogrel in comparison to aspirin led to fewer thrombotic events in patients who were post-MI, post-stroke, or had peripheral arterial disease (PAD)
2001	CURE	Clopidogrel	Addition of clopidogrel to aspirin resulted in 2% reduction in the risks for cardiovascular events including MI, stroke (MACCE), although with a 1% increase in major bleeding
2001	PCI CURE	Clopidogrel	Initiation of clopidogrel before PCI and its continuation for a mean of 8 months after PCI with stent implantation, along with aspirin provided considerable mortality benefit without significant increase in bleeding
2002	CREDO	Clopidogrel	Prolonged therapy with clopidogrel after PCI reduced the risk for death, MI and stroke by 3% at 1 year after randomization
2007	TRITON TIMI 38	Prasugrel	In patients with ACS and scheduled PCI, prasugrel demonstrated superior efficacy compared to clopidogrel in reducing ischemic events including stent thrombosis but with significantly higher bleeding
2009	PLATO	Ticagrelor	In patients with ACS, ticagrelor reduced rates of cardiovascular(CV) death, MI, or stroke and all-cause mortality at 12 months in comparison with clopidogrel without a significant difference in major bleeding. There was more non-CABG-related bleeding in the ticagrelor group. These benefits were less prominent in the USA cohort. The 2012 post-hoc analysis accounted for only aspirin dose for such a difference (2012-mahaffey)
2012	TRILOGY ACS	Prasugrel	Among patients with unstable angina (UA) or myocardial infarction without ST-segment elevation (NSTEMI), prasugrel did not significantly reduce the frequency of CV death), MI, or stroke, as compared with clopidogrel, and similar risks of bleeding were observed.
2013	CHAMPION PHOENIX	Cangrelor	Potent intravenous adenosine diphosphate (ADP) receptor antagonist was evaluated in patients undergoing elective or urgent PCI in comparison with standard therapy. There were 1.2% fewer MACCE events including ST in cangrelor arm without any significant increase in severe bleeding
2012	TRACER investigators	Vorapaxar	Oral protease-activated-receptor 1 (PAR-1) antagonist that inhibits thrombin-induced platelet activation was evaluated in patients with ACS. There was no significant reduction in MACCE but it accounted for significant increase in the risk of major bleeding, including intracranial hemorrhage (ICH). Later, it was shown to reduce MACCE by about 1.2% in comparison with standard therapy in stable patients but at the cost of increased risk of moderate or severe bleeding including ICH

MACCE = major adverse cardiovascular and cerebrovascular events; ICH = intracranial hemorrhage. ISIS-2 [25] = Second International Study of Infarct Survival Collaborative Group; CAPRIE [26] = a randomized, blinded, trial of Clopidogrel versus Aspirin in Patients at Risk of Ischemic Events; CURE [27] = Clopidogrel in Unstable angina to prevent Recurrent Events; PCI-CURE [28] = Effects of pretreatment with clopidogrel and aspirin followed by long-term therapy in patients undergoing percutaneous coronary intervention; CREDO [29] = The Clopidogrel for the Reduction of Events During Observation; TRITON-TIMI 38 [30] = TRial to assess Improvement in Therapeutic Outcomes by optimizing platelet Inhibition with Prasugrel–Thrombolysis in Myocardial Infarction, PLATO [31] = Platelet inhibition And patient Outcomes; TRILOGY ACS [32] = The TaRgeted platelet Inhibition to cLarify the Optimal strateGy to medicallY manage Acute Coronary Syndromes; CHAMPION PHOENIX [33] = Effect of platelet inhibition with cangrelor during PCI on ischemic events; TRACER [34] = Thrombin-Receptor Antagonist for Clinical Event Reduction.

**Table 3:** Comparative features of randomized controlled trials (RCTs) for short DAPT (S-DAPT) vs. standard DAPT.

Trial	Year	Randomization of DAPT Duration (Months)	Number of Patients (Group 1; Group 2)	Placebo Control	ACS (%)	DM (%)	1G DES (%)	2G DES (%)	Primary Endpoint	Event Rate Intervention vs. Control (Noninferiority Margin)
EXCELLENT	2012	6 vs. 12 (noninferiority)	1443 (722; 721)	No	52	38	25	75	Cardiac death, MI, TVR	4.8% vs. 4.3% *p*_ni_ = 0.001 (4%)
RESET	2012	3 vs. 12 (noninferiority)	2117 (1059; 1058)	No	54	29	21	85	Cardiac death, MI, TVR, ST, TIMI major or minor bleeding	4.7% vs. 4.7% *p*_ni_ < 0.001
OPTIMIZE	2013	3 vs. 12 (noninferiority)	3119 (1563;1556)	No	32	35	-	100	Death, MI, stroke, major bleeding	6% vs. 5.8% *p*_ni_ = 0.002
SECURITY	2014	6 vs. 12 (noninferiority)	1399 (682; 717)	No	38	31	-	100	Cardiac death, MI, ST, stroke, BARC 3/5 bleeding	4.5% vs. 3.7% *p*_ni_ < 0.05 (2%)
ISAR-SAFE	2015	6 vs. 12 (noninferiority)	4000 (1997; 2003)	Yes	40	25	10	89	Death, MI, ST, stroke, TIMI major bleeding	1.5% vs. 1.6% *p*_ni_ <0.001 (2%)
I LOVE IT	2016	6 vs. 12 (noninferiority)	1829	No	82	-	-	-	Cardiac death, MI, TLR	6.8% vs. 5.9% *p*_ni_ < 0.05 (3.7%)
IVUS XPL	2016	6 vs. 12 (comparability)	1400	No	49	-	-	-	Cardiac death, MI, stroke, TIMI major bleeding	2.2% vs. 2.1% *p* = 0.85
DAPT-STEMI	2017	6 vs. 12 (noninferiority)	1100	No	100	14	-	100	All cause mortality, MI, revascularization, stroke, and TIMI major bleeding	4.8% vs. 6.6% *p*_ni_ = 0.004
REDUCE	2017	3 vs. 12 (noninferiority	1496	No	100	-	-	100	Death, MI, stroke and bleeding	8.2% vs. 8.4% *p*_ni_ < 0.01

ACS = acute coronary syndrome; DM = diabetes mellitus; 1G = first generation; 2G = second generation; MI = myocardial infarction; TVR = target vessel revascularization; *p*_ni_ = *p* value for noninferiority; ST = stent thrombosis; TIMI = thrombolysis in myocardial infarction; BARC = bleeding academic research consortium; TLR = target lesion revascularization. EXCELLENT [39] = Six-month versus 12-month dual antiplatelet therapy after implantation of drug-eluting stents: the Efficacy of Xience/Promus versus Cypher to REduce Late Loss After Stenting randomized, multicenter study; RESET [40] = REal Safety and Efficacy of 3-month dual antiplatelet Therapy following Endeavor zotarolimus-eluting stent implantation; OPTIMIZE [41] = Three vs. twelve months of dual antiplatelet therapy after zotarolimus-eluting stents; SECURITY [42] = Second-generation drug-eluting stent implantation followed by 6- versus 12-month dual antiplatelet therapy; ISAR-SAFE [43] = Intracoronary Stenting and Antithrombotic Regimen: Safety and Efficacy of 6 months’ DAPT after DES; I LOVE IT [44] = a randomized, double-blind, placebo-controlled trial of 6 vs. 12 months of clopidogrel therapy after drug-eluting stenting; IVUS XPL [45] = 6-Month Versus 12-Month Dual-Antiplatelet Therapy Following Long Everolimus-Eluting Stent Implantation; DAPT-STEMI [46] = A prospective, randomized, open-label trial of 6-month versus 12-month dual antiplatelet therapy after drug-eluting stent implantation in ST-elevation myocardial infarction; REDUCE [47] = Randomized evaluation of short-term dual antiplatelet therapy in patients with acute coronary syndrome treated with the COMBO dual therapy stent.

**Table 4:** Comparative featurmmmes of randomized controlled trials (RCTs) for short DAPT (S-DAPT) vs. long DAPT (L-DAPT).

Trial	Year	Randomization of DAPT Duration (Months)	Number of Patients (Group 1; Group 2)	Placebo Control	ACS (%)	DM (%)	1G DES (%)	2G DES (%)	Primary Endpoint	Event Rate (Intervention vs. Control)
PRODIGY	2012	6 vs. 24 (superiority of 24 months) 25% BMS	1970	No	75	24	25	50	Death, MI, stroke	10% vs. 10.1% *p* = 0.91
ITALIC	2015	6 vs. 24 (noninferiority of 6 months)	1822	No	24	37	-	100	Death, MI, stroke, TVR, major bleeding	1.6% vs. 1.5% *p*_ni_ = 0.0002
NIPPON	2017	6 vs. 18 (noninferiority of 6 months)	3773	No	28	33	-	100	Death, MI, stroke, major bleeding	2.1% vs. 1.5%

ACS = acute coronary syndrome; DM = diabetes mellitus; 1G = first generation; 2G = second generation; MI = myocardial infarction; TVR = target vessel revascularization; *p*_ni_ = *p* value for noninferiority. PRODIGY [48] = The PROlonging Dual AntIplatelet Treatment After Grading Stent-Induced Intimal Hyperplasia Study; short-versus long-term duration of dual-antiplatelet therapy after coronary stenting: a randomized multicenter trial; ITALIC [49] = Is There A Life for DES after DIscontinuation of Clopidogrel, 6- versus 24-month dual antiplatelet therapy after implantation of drug-eluting stents in patients nonresistant to aspirin; NIPPON [50] = Dual Antiplatelet Therapy for 6 Versus 18 Months After Biodegradable Polymer Drug-Eluting Stent Implantation.

**Table 5:** Comparative features of randomized controlled trials (RCTs) for standard DAPT Vs. long DAPT (L-DAPT).

Trial	Year	Randomization of DAPT Duration (Months)	Number of Patients	Placebo Control	ACS (%)	DM (%)	1G DES (%)	2G DES (%)	Primary Endpoint	Event Rate (Intervention vs. Control)
DAPT	2014	12 vs. 30	9961	yes	43	31	38	60	Death, MI, stroke, and definite/probable ST	ST–1.4% vs. 0.4%; *p* < 0.0001, NNT = 100 MACCE-5.9% vs. 4.3%; *p* < 0.001, NNT = 62
DES LATE	2014	12 vs. 36	5045	no	61	28	64	30	Cardiac death, MI, stroke	2.4% vs. 2.6% *p* = 0.75
ARCTIC INTERRUPTION	2014	12 vs. 18–24	1259	no	-	34	40	60	Death, MI, stroke, ST, TVR	4% vs. 4% *p* = 0.58
OPTIDUAL	2016	12 vs. 14–48	1385	yes	36	31	34	66	Death, MI, stroke, ISTH major bleeding	7.5% vs. 5.8% *p* = 0.17

ACS = acute coronary syndrome; DM = diabetes mellitus; 1G = first generation; 2G = second generation; MI = myocardial infarction; TVR = target vessel revascularization; *p*_ni_ = *p* value for noninferiority; ST = stent thrombosis; MACCE = major adverse cardiovascular and cerebrovascular events; ISTH = international society of thrombosis and hemostasis. DAPT [23] = Dual AntiPlatelet Therapy study, Twelve or 30 months of dual antiplatelet therapy after drug-eluting stents; DES LATE [51] = Optimal duration of dual antiplatelet therapy after drug-eluting stent implantation; ARCTIC INTERRUPTION [52] = Dual-antiplatelet treatment beyond 1 year after drug-eluting stent implantation; OPTIDUAL [53] = Stopping or continuing clopidogrel 12 months after drug-eluting stent placement.

**Table 6:** Guideline statements on DAPT usage.

Disease State	ESC 2017 DAPT	ACC/AHA 2016 DAPT
ACS (Medical therapy, BMS or DES)	12 months (I-A) 6 months in patients with high bleeding risk (II a-B) >12 months may be considered in patients with prior MI at low bleeding risk (II b-B)	At least 12 months (I-B) 6 months in patients with high bleeding risk (II b-C) >12 months may be reasonable in patients at low bleeding risk (II b-A)
Stable CAD and BMS	6 months (I-A) In patients with high bleeding risk, 1 month (II b-C) or 3 months (II a-B)	At least 1 month (I-A)
Stable CAD and DES	6 months (I-A) In patients with high bleeding risk, 1 month (II b-C) or 3 months (II a-B)	At least 6 months (I-B)

ESC = European Society of Cardiology focused update on dual antiplatelet therapy in coronary artery disease [3]. ACC/AHA = American College of Cardiology/American Heart Association guideline focused update on duration of dual antiplatelet therapy in patients with coronary artery disease [2].

**Table 7:** Comparative features of tools for risk estimation.

	DAPT Score	PRECISE-DAPT Score
Risk assessed	Combined bleeding and ischemia	Bleeding
Variables	Age (65–74: −1, >75: −2), DM (+1), prior MI/PCI (+1), ACS (+1), stent diameter < 3 mm (+1), LVEF < 30% (+1), Vein graft stent (+2), tobacco use (+1)	Hemoglobin, age, prior bleeding, creatinine clearance
Time to use	After 12 months of uneventful DAPT	At the time of coronary stenting
Cessation strategies assessed	Standard DAPT vs. L-DAPT (>30 months)	S-DAPT (3–6 months) vs. standard/L-DAPT (12–24 months)
Score range (points)	−2 to 10	0 to 100
Decision suggested based on score in points	L-DAPT for score ≥ 2 Standard DAPT for score < 2	S-DAPT for score ≥ 25 Standard/ L-DAPT < 25
Online resource	http://tools.acc.org/DAPTriskapp/#!/content/calculator/	www.precisedaptscore.com

DM = diabetes mellitus; MI = myocardial infarction; PCI = percutaneous coronary intervention, ACS = acute coronary syndrome; LVEF = left ventricular ejection fraction.

**Table 8:** Evidence on triple therapy.

Trial	Number of Patients	Randomization	Outcome
WOEST	573	Patients randomized to receive triple therapy with aspirin (80 mg/day) + clopidogrel and warfarin vs. clopidogrel and warfarin-1 month for BMS; 1 year for DES	TIMI bleeding at 1 year was significantly reduced in dual therapy arm (19.5% vs. 44.4%; HR 0.36, 95% CI 0.26–0.50, *p* < 0.001), and lower all-cause mortality (2.5% vs. 6.4%; *p* = 0.027) No significant differences in major bleeding, MI, stroke, TVR, ST.
PIONEER AF-PCI	2124	1:1:1 design in patients with non valvular AF and PCI to low-dose rivaroxaban (15 mg daily) + P2Y_12_ inhibitor for 12 months; very low dose rivaroxaban (2.5 mg BID) +DAPT for 1, 6, or 12 months or standard therapy with dose adjusted warfarin + DAPT for 1, 6, or 12 months	Both rivaroxaban groups had lower primary safety endpoint vs. standard therapy (16.8%, 18% vs. 26.7%; with HR 0.59, 95% CI 0.47–0.76, *p* < 0.0001 and HR 0.63, 95% CI 0.5–0.8, *p* < 0.001 respectively)
REDUAL-PCI	2725	Random assignment of patients with AF who had undergone PCI to either triple therapy or dual therapy. The triple therapy group received warfarin, plus a P2Y inhibitor (clopidogrel or ticagrelor) and aspirin (for 1–3 months), while the dual therapy group received dabigatran (110 mg or 150 mg twice daily) plus a P2Y inhibitor (clopidogrel or ticagrelor)	Primary endpoint of major or clinically relevant nonmajor bleeding event during the 14-month follow up period was lower in both dabigatran groups in comparison with triple therapy. 110 mg dual therapy group vs. triple therapy group (15.4% vs. 26.9%, *p*_ni_ < 0.001) 150 mg dual therapy group vs. triple therapy group (20.2% vs. 25.7%, *p*_ni_ < 0.001) The primary efficacy outcome, incidence of death, MI, stroke, systemic embolism, or unplanned revascularization, occurred in 13.7% of the two dual therapy groups vs. 13.4% of the triple therapy group (*p*_ni_ = 0.005) The rate of serious adverse events did not vary significantly among the groups.

BMS = bare metal stent; DES = drug eluting stent; TIMI = thrombolyisis in myocardial infarction; HR = hazard ratio, CI = confidence interval; MI = myocardial infarction; TVR = target vessel revascularization, BID = twice daily, AF = atrial fibrillation. WOEST [80] = What is the Optimal antiplatElet and anticoagulant therapy in patients with oral anticoagulation and coronary StenTing; PIONEER AF-PCI [81] = Prevention of Bleeding in Patients with Atrial Fibrillation Undergoing PCI; REDUAL-PCI [82] = Dual Antithrombotic Therapy with Dabigatran after PCI in Atrial Fibrillation.

**Table 9:** Recommended therapeutic strategies for patients needing anticoagulation and anti-platelet therapy.

DAPT Strategy	Higher Ischemia Risk	Higher Bleeding Risk
Initial	1 month (IIa-B) or 6 month (IIa-B) triple therapy	1 month triple therapy (IIa-B) Or 12 months dual therapy with oral anticoagulant and clopidogrel (IIa-A)
Continuation	Up to 12 month therapy with oral anticoagulant & aspirin/clopidogrel (IIa-A) Oral anticoagulant alone beyond 12 months (IIa-B)	Up to 12 months dual therapy with oral anticoagulant & aspirin/clopidogrel (IIa-A) Oral anticoagualnt alone beyond 12 months (IIa-B)

Adapted from ESC guideline statement [3].

**Table 10:** Comparative features of prominent future randomized controlled trials evaluating novel DAPT regimens.

Trial	Expected Completion	Hypothesis	Randomization	Stent Type	Primary Endpoint
GLOBAL LEADERS	2017	Superiority of 1 month DAPT followed by SAPT with ticagrelor vs. 12 months DAPT followed by SAPT with aspirin	1 month DAPT followed by ticagrelor for 23 months vs. 12 months DAPT followed by aspirin	Biodegradable polymer biolimus A9-eluting stent	Death or MI
MASTER DAPT	2019	Noninferiority of 1 month DAPT vs. 3/6 months	1 month vs. 3–6 months	Biodegradable polymer sirolimus eluting stent	Death, MI, stroke, and BARC 3/5 bleeding
TWILIGHT	2019	Superiority of 3 months DAPT followed by ticagrelor alone vs. 15 months DAPT	3 months DAPT followed by ticagrelor alone for 12 months vs. 15 months DAPT with aspirin and ticagrelor	DES	BARC 2, 3, or 5 bleeding

MI = myocardial infarction; BARC = bleeding academic research consortium. GLOBAL LEADERS = A Clinical Study comparing two forms of antiplatelet therapy after stent implantation (NCT01813435); MASTER DAPT = Management of high bleeding risk patients post bioresorbable polymer coated stent implantation with an abbreviated versus prolonged DAPT regimen (NCT03023020); TWILIGHT = Ticagrelor with Aspirin or alone in high-risk patients after coronary intervention (NCT02270242).
